# Supplementary material for: Comparative transcriptomics in three Methylophilaceae species uncover different strategies for environmental adaptation
Source: PeerJ. 2013 Jul 25;1:e115. doi: 10.7717/peerj.115 (PMC3728764; doi:10.7717/peerj.115)
Supplement: Table S1 [file peerj-01-115-s001.docx]

**Supplemental Table 1. RNA-Seq statistics**

| Sample | Total reads (millions) | Reads mapped (millions) | Reads mapped (%) | Reads mapped to CDS (millions) | Reads mapped to CDS (%) | Replicate agreement (R) |
| --- | --- | --- | --- | --- | --- | --- |
| 1 (JLW8, MA) | 16.8 | 15.9 | 94.7 | 0.8 | 4.9 | .84 |
| 2 (JLW8, MA) | 27.9 | 27.0 | 96.7 | 2.4 | 8.9 |  |
| 3 (JLW8, Mei) | 9.3 | 9.3 | 95.8 | 0.7 | 7.3 | .97 |
| 4 (JLW8, Mei) | 12.1 | 12.1 | 95.8 | 0.9 | 8.1 |  |
| 5 (JLW8, Core) | 21.9 | 19.7 | 89.9 | 0.4 | 1.8 | .95 |
| 6 (JLW8, Core) | 32.9 | 32.4 | 98.4 | 0.4 | 1.3 |  |
| 7 (301, MA) | 175.0 | 174.3 | 99.6 | 8.7 | 5.0 | .98 |
| 8 (301, MA) | 184.9 | 184.2 | 99.6 | 2.3 | 1.2 |  |
| 9 (301, Me-i) | 185.8 | 154.4 | 83.1 | 1.9 | 1.2 | .99 |
| 10 (301, Me-i) | 169.8 | 127.6 | 75.1 | 11.5 | 9.0 |  |
| 11 (301, Core) | 24.0 | 21.7 | 90.7 | 0.1 | 0.5 | .92 |
| 12 (301, Core) | 37.5 | 37.3 | 99.4 | 0.1 | 0.4 |  |
| 13 (SIP3-4, MA) | 190.5 | 175.4 | 92.1 | 17.4 | 9.9 | 98 |
| 14 (SIP3-4, MA) | 157.7 | 143.8 | 91.2 | 9.5 | 6.6 |  |
| 15 (SIP3-4, Me) | 114.0 | 113.0 | 99.1 | 0.2 | 0.2 | .98 |
| 16 (SIP3-4, Me) | 112.8 | 111.8 | 99.1 | 0.2 | 0.2 |  |
| 17 (SIP3-4, Core) | 36.2 | 36.0 | 99.4 | 0.7 | 1.8 | .96 |
| 18 (SIP3-4, Core) | 35.8 | 35.4 | 98.8 | 0.6 | 1.7 |  |
