# Supplementary material for: Comparative transcriptomics in three Methylophilaceae species uncover different strategies for environmental adaptation
Source: PeerJ. 2013 Jul 25;1:e115. doi: 10.7717/peerj.115 (PMC3728764; doi:10.7717/peerj.115)
Supplement: Table S11 — Gene designations, functions and protein identifiers for key enzyme systems known to be or potentially involved in methylotrophy. [file peerj-01-115-s011.docx]

**Supplemental Table 11.** Gene designations, functions and protein identifiers for key enzyme systems known to be or potentially involved in methylotrophy

Gene name Protein/ function Protein identifiers JLW8 301 SIP3-4

***Methanol oxidation***

*mxaF* MDH large subunit - - Msip34_0734

*mxaJ*  essential for MDH function - - Msip34_0735

*mxaG* cytochrome, electron acceptor from MDH - - Msip34_0736

*mxaI* MDH small subunit - - Msip34_0737

*mxaR* chaperonin Mmol_1107 M301_1093 Msip34_0738

Mmol_1541 M301_2233 Msip34_0870

- - Msip34_2692

*mxaS*  unknown Mmol_1106 M301_1092 Msip34_0739

Mmol_1542 M301_2232 Msip34_0869

- - Msip34_2693

*mxaA* essential for Ca^2+^ insertion into MDH Mmol_1105 M301_1091 Msip34_0740

Mmol_1543 M301_2231 Msip34_0868

- - Msip34_2694

*mxaC* essential for Ca^2+^ insertion into MDH Mmol_1104 M301_1090 Msip34_0741

Mmol_1544 M301_2230 Msip34_0867

- - Msip34_2695

*mxaK* essential for Ca^2+^ insertion into MDH Mmol_1103 M301_1089 Msip34_0742

Mmol_1545 M301_2229 Msip34_0866

- - Msip34_2696

*mxaL* essential for Ca^2+^ insertion into MDH Mmol_1102 M301_1088 Msip34_0743

Mmol_1546 M301_2228 Msip34_0865

- - Msip34_2697

*mxaD* unknown - - Msip34_0744

*xoxF* MxaF homolog Mmol_1770 M301_1208 Msip34_0016

Mmol_2048 M301_2463 Msip34_2348

- - Msip34_2549

- M301_1659 Msip34_1813

*xoxJ* MxaJ homolog Mmol_2047 M301_2462 Msip34_2347

*xoxG* MxaG homolog Mmol_2045 M301_2459 Msip34_2346

Mmol_2046 - -

- M301_2460 -

- M301_2461 -

- - Msip34_0015

- - Msip34_2345

***Pyrroloquinoline quinone (PQQ) biosynthesis***

*pqqA* PQQ synthesis Mmol_0021 M301_0054 Msip34_0020

Mmol_0459 M301_1191 Msip34_1529

Mmol_0794 M301_2123 Msip34_2759

Mmol_0993 M301_2651 Msip34_2777

*pqqB* PQQ synthesis Mmol_0994 M301_1192 Msip34_1528

*pqqC* PQQ synthesis Mmol_0995 M301_1193 Msip34_1527

*pqqD* PQQ synthesis Mmol_0996 M301_1194 Msip34_1596

*pqqE* PQQ synthesis Mmol_0997 M301_1195 Msip34_1525

*pqqF* PQQ synthesis Mmol_1750 M301_2041 Msip34_1921

*pqqG* PQQ synthesis Mmol_1751 M301_2042 Msip34_1922

***Methylamine oxidation (methylamine dehydrogenase, MADH)***

*mauF* unknown Mmol_1576 - -

*mauB*  MADH large subunit Mmol_1575 - -

*mauE* essential for small subunit maturation Mmol_1574 - -

*mauD* essential for small subunit maturation Mmol_1573 - -

*mauA* MADH small subunit Mmol_1572 - -

*mauG* TTQ biosynthesis Mmol_1571 - -

*mauL* unknown Mmol_1570 - -

*mauM* unknown Mmol_1569 - -

*mauN* unknown Mmol_1568 - -

*mauO* cytochrome Mmol_1567 - -

***Methylamine oxidation (N-methylglutamate pathway)***

*mgdA* N-methylglutamate DH sub. A - M301_1421 Msip34_2428

*mgdB* N-methylglutamate DH sub. B - M301_1420 Msip34_2427

*mgdC* N-methylglutamate DH sub. C - M301_1419 Msip34_2426

*mgdD* N-methylglutamate DH sub. D - M301_1418 Msip34_2425

gma g-glutamylmethylamide synthase - M301_1417 Msip34_2424

*mgsA* N-methylglutamate synthase sub. A - M301_1416 Msip34_2423

*mgsB* N-methylglutamate synthase sub. B - M301_1415 Msip34_2422

*mgsC* N-methylglutamate synthase sub. C - M301_1414 Msip34_2421

***H_4_MPT-linked formaldehyde oxidation***

*fhcB* Fhc, subunit B Mmol_0862 M301_0913 Msip34_1506

*fhcA* Fhc, subunit A Mmol_0861 M301_0912 Msip34_1507

orf FMN-binding protein Mmol_0860 M301_0911 -

*fhcD* Fhc, subunit D Mmol_0859 M301_0910 Msip34_1508

*fhcC* Fhc, subunit C Mmol_0858 M301_0909 Msip34_1509

*mptG* β-RFAP synthase Mmol_1347 M301_1557 Msip34_1505

*mtdB* methylene H_4_MPT dehydrogenase Mmol_1346 M301_1556 Msip34_1504

*orfY* unknown Mmol_1345 M301_1555 Msip34_1503

*mch* methenyl H_4_MPT cyclohydrolase Mmol_1344 M301_1554 Msip34_1502

*orf5* biosynthesis of H_4_MPT Mmol_1343 M301_1553 Msip34_1501

*orf7*  unknown Mmol_1342 M301_1552 Msip34_1500

*fae*  formaldehyde activating enzyme Mmol_1253 M301_0896 Msip34_1495

Mmol_2056 M301_1343 Msip34_1496

*orf17* unknown Mmol_1336 M301_1545 Msip34_1494

*orf1* unknown Mmol_1335 M301_1544 Msip34_1493

*orf9* biosynthesis of H_4_MPT Mmol_1334 M301_1543 Msip34_1492

*pabB* para-aminobenzoate synthase component I Mmol_1333 M301_1542 Msip34_1491

*orf21* biosynthesis of H_4_MPT Mmol_1332 M301_1541 Msip34_1490

*pcbD* unknown Mmol_1331 M301_1540 Msip34_1489

*orf22* biosynthesis of H_4_MPT Mmol_0899 M301_0953 Msip34_1682

*orf19* biosynthesis of H_4_MPT Mmol_0898 M301_0952 Msip34_1683

*orf20* biosynthesis of H_4_MPT Mmol_0897 M301_0951 Msip34_1684

*afp* dihydromethanopterin reductase Mmol_0896 M301_0950 Msip34_1685

*fae2* formaldehyde activating enzyme homolog Mmol_0024 M301_2758 Msip34_2608

*fae3*  formaldehyde activating enzyme homolog - M301_1423 Msip34_2416

***Formate oxidation***

*fdh1A* FDH1, dehydrogenase alpha subunit Mmol_2033 M301_2447 Msip34_1179

*fdh1B* FDH1, beta subunit Mmol_2034 M301_2448 Msip34_1178

*fdh1C* FDH1, gamma subunit Mmol_2035 M301_2449 Msip34_1177

*fdh1D* FDH1, accessory protein Mmol_2032 M301_2446 Msip34_1180

*fdh1E* FDH1, delta subunit Mmol_2031 M301_2445 Msip34_1181

*fdh4A* FDH4, dehydrogenase alpha subunit Mmol_0470 - Msip34_1600

*fdh4B* FDH4-associated protein Mmol_0469 - Msip34_1599

***Ribulose monophosphate cycle for formaldehyde assimilation/oxidation***

*hps1* hexulosephosphate synthase Mmol_0313 M301_0304 Msip34_0268

*hps2* Mmol_1338 M301_1547 Msip34_1498

*hpi* hexulosephosphate isomerase Mmol_1337 M301_1546 Msip34_1497

*tal* transaldolase Mmol_1339 M301_1548 Msip34_1499

*pgi* glucose 6-phosphate isomerase Mmol_0827 M301_1501 Msip34_1269

*zwf* glucose 6-phosphate dehydrogenase Mmol_1527 M301_1112 Msip34_1093

*pgl* 6-phosphogluconolactonase Mmol_1526 M301_1114 Msip34_1095

*gndA* 6-phosphogluconate dehydrogenase (NAD) - - Msip34_1094

*gndB* 6-phosphogluconate dehydrogenase (NADP) Mmol_0143 M301_2666 -

*edd* 6-phosphogluconate dehydratase Mmol_1727 M301_2019 Msip34_1897

*eda* 2-keto 3-deoxy 6-phosphogluconate aldolase Mmol_1726 M301_2018 Msip34_1896

*tkt* transketolase Mmol_1980 M301_2400 Msip34_0483

*rpe* ribulosephosphate 3-epimerase Mmol_2239 M301_2582 Msip34_2516

*ppi* ribose 5-phosphate isomerase Mmol_0287 M301_0182 Msip34_0164

Mmol_1429 M301_1566 Msip34_1138

***Methylcitric acid cycle***

*prpD* Methylcitrate dehydratase Mmol_0748 M301_0686 -

*prpC* Methylcitrate synthase Mmol_0749 M301_0687 -

*prpB* Methylisocitrate lyase Mmol_0750 M301_0688 -

*cyl1* Citrate lyase-like Mmol_0751 M301_0689 -

*maoC* Dehydratase Mmol_0752 M301_0690 -

*cyl2* Citrate lyase-like Mmol_0753 M301_0691 -

*gntR* Transcriptional regulator Mmol_0754 M301_0692 -

*mdh* Malate dehydrogenase Mmol_0756 M301_0693 Msip34_2203

*sdhC* Succinate dehydrogenase, C subunit Mmol_0757 M301_0694 -

*sdhD* Succinate dehydrogenase, D subunit Mmol_0758 M301_0695 -

*sdhA* Succinate dehydrogenase, A subunit Mmol_0759 M301_0696 -

*sdhB* Succinate dehydrogenase, B subunit Mmol_0760 M301_0697 -

*orf* Hypothetical protein Mmol_0761 M301_0698 -

*cyl3* Citrate lyase-like Mmol_0762 M301_0699 -

*prpE* Propionyl-CoA synthase Mmol_0763 M301_0700 -

*fum* Fumarase Mmol_0764 M301_0701 -

*orf* Hypothetical protein Mmol_0765 M301_0702

*acn* Aconitate hydratase Mmol_0766 M301_0703 -

MDH, methanol dehydrogenase; H_4_MPT, tetrahydromethanopterin; β-RFAP, β-ribofuranosylaminobenzene.
